# Electrophysiological and neuropsychological assessment of cognition in spinocerebellar ataxia type 1 patients: a pilot study

**DOI:** 10.1007/s10072-022-06597-5

**Published:** 2023-01-14

**Authors:** Elena Contaldi, Mariachiara Sensi, Fabiana Colucci, Jay Guido Capone, Arianna Braccia, Mattia Roberto Nocilla, Enrica Diozzi, Eleonora Contini, Anna Chiara Pelizzari, Valeria Tugnoli

**Affiliations:** 1 PhD Program in Medical Sciences and Biotechnology, University of Piemonte Orientale, 28100 Novara, Italy; 2 Department of Neuroscience and Rehabilitation, University of Ferrara, 44124 Ferrara, Italy; 3 Movement Disorders Centre, Neurology Unit, Department of Translational Medicine, University of Piemonte Orientale, Corso Mazzini 18, 28100 Novara, Italy; 4 Department of Neuroscience and Rehabilitation, Azienda Ospedaliero-Universitaria S. Anna, 44124 Ferrara, Italy

**Keywords:** Spinocerebellar ataxia type 1, SCA1, Cerebellar cognitive affective syndrome, Auditory event-related potentials, N100, N200, P300

## Abstract

**Background:**

Event-related potentials (ERPs) reflect cognitive processing: negative early components (N100, N200) are involved in the sensory and perceptual processing of a stimulus, whereas late positive component P300 requires conscious attention. Both neuropsychological and affective disorders are present in patients with spinocerebellar ataxia type 1 (SCA1), but the underlying mechanisms need further clarification.

**Materials and methods:**

In this pilot study, we assessed cognitive processing by recording auditory ERPs in 16 consecutive SCA1 patients and 16 healthy controls (HC) matched for age and sex. Motor and nonmotor symptoms were evaluated using the Scale for the Assessment and Rating of Ataxia (SARA) and an extensive neuropsychological battery. ERPs were recorded using an oddball paradigm, and peak latency and amplitude of N100, N200, and P300 were measured in the averaged responses to target tones.

**Results:**

We found in SCA1 significantly increased latencies of N200 and P300 (p=0.033, p=0.007) and decreased amplitudes of N100 and P300 (p=0.024, p=0.038) compared with HC. Furthermore, P300 latency had the highest AUC in the discrimination of SCA1 in ROC analysis. The expansion of trinucleotide repeats correlated with P300 latency (r=−0.607, p=0.048), whereas both P300 and N100 amplitudes correlated with the severity of motor symptoms (r=−0.692, p=0.003; r=−0.621; p=0.010). Significant correlations between P300 latency and the scores of Emotion Attribution Task (r=−0.633, p=0.027), as well as between N200 latency and the scores of Frontal Assessment Battery and Stroop test (r=−0.520, p=0.047; r=0.538, p=0.039), were observed.

**Conclusions:**

This research provides for the first time an extensive characterization of ERPs as useful electrophysiological markers to identify early cognitive dysfunction in SCA1.

**Supplementary Information:**

The online version contains supplementary material available at 10.1007/s10072-022-06597-5.

## Introduction

The spinocerebellar ataxias (SCAs) are a heterogeneous group of neurodegenerative disorders characterized by the main involvement of cerebellar functions and an autosomal dominant inheritance. Both repeat expansions and non-repeat mutations have been described, mostly causing damage to cerebellar Purkinje neurons, even though other parts of the central and peripheral nervous system can be involved [1]. Spinocerebellar ataxia type 1 (SCA1) represents 3–16 % of all SCAs and has a global prevalence of 1–2 in 100,000 with significant geographical variations [2]. Along with cerebellar motor deterioration, cognitive and affective functions may also be affected, contributing to the development of the cerebellar cognitive affective syndrome (CCAS) described by Schmahmann in 1998 [3]. The involvement of executive functions, orientation, and logical reasoning was observed by Ma et al. [4]. Moriarty and colleagues further reported in different subtypes of SCAs decreased function in the executive, visual memory, and social-affective domains, finding the most rapid cognitive decline in SCA1 patients [5]. Since SCA1 is a multi-domain neurodegenerative disorder, several studies explored the integrity of different pathways using neurophysiology, reporting abnormalities in visual, motor, somatosensory, and brainstem auditory evoked potentials [6]. Nonetheless, less is known about endogenous event-related potentials (ERPs) in SCAs. ERPs are known to primarily provide valuable information about attention and memory function: in the “oddball” paradigm subjects respond only to a specific target stimulus randomly presented with non-target stimuli, which implies the updating of working memory and the relocation of attentive resources. ERPs mirror cognitive processing from the evaluation of earlier sensory stimuli to later cognitive components requiring attentional resources: early components reflect sensory and perceptual processing of a stimulus and occur without or before conscious attention from the participant, whereas later components require conscious attention. The N100 reflects a change in auditory stimulation, like the offset and onset of sounds [7]. Typically evoked 190 and 360 ms after the presentation of a specific visual or auditory stimulus, the N200 is a fronto-central negative wave reflecting cognitive processes of stimulus evaluation and resulting from a deviation from a prevailing stimulus [8]. In the context of an oddball paradigm, the mismatch negativity (MMN), or auditory N2a, is elicited in a task-independent manner and reflects the discrimination between the deviating stimulus and the sensory-memory representation of the standard stimulus [9]. This component is independent of attention and top-down processes and can be found in both comatose patients and unresponsive wakefulness syndrome [10]. The P300 is a later (typically >250 ms) component, believed to be an index of active cognitive processing, especially attention and memory processes [11]. Different generators have been identified including the frontal and parietal cortex, amygdala, hippocampus, parahippocampal gyrus, temporal lobe, and basal ganglia. It requires the participant to pay attention to all the stimuli and categorize the difference between the standard and the rare target stimuli [11]. All ERP waveforms can be quantitatively characterized in amplitude and latency: the amplitude depends on the amount of neural activation, whereas the latency reveals the timing of this activation.

ERPs have been widely employed to investigate brain function in several disorders, i.e., Alzheimer’s disease (AD) and mild cognitive impairment (MCI) [12, 13]. Recently, Rodrìguez-Labrada and colleagues explored in a cross-sectional study visual and auditory ERPs in 30 SCA2 patients, 20 preclinical carriers, and 33 healthy controls (HC) [14]: compared with HC, patients had increased P300 latencies and decreased P300 amplitudes, whereas preclinical carriers exhibited only a prolongation of P300 latencies. Intriguingly, visual P300 latencies were significantly associated with time-to-ataxia onset in preclinical carriers, thus identifying this measure as a feasible biomarker of the prodromal stage of the disease [14].

Even though previous research highlighted progressive cognitive dysfunction in SCA1 patients, the role of auditory ERPs is not yet fully elucidated. With the present study, we aim to provide a thorough characterization of auditory ERP components (N100, N200, and P300) in SCA1 compared with HC and explore the relationship between ERPs and motor/cognitive measures.

## Methods

### Subjects

Genetically determined SCA1 patients referring to our outpatient clinic for movement disorders were consecutively examined for eligibility criteria. Demographic and clinical data were collected, and SCA1 patients were evaluated for motor symptoms and global cognition by neurologists with experience in movement disorders through the Scale for the Assessment and Rating of Ataxia (SARA) [15] and Mini-Mental State Examination (MMSE) [16]. As exclusion criteria, we considered: severe cerebellar impairment with SARA score > 24 or cognitive impairment with MMSE < 24 (to maximize adherence to the study protocol), and a history of hearing loss. The initial cohort consisted of 17 patients (9 males and 8 females), but one subject was excluded from the study because ERP components were poorly discernible. Therefore, the final analysis was performed on *N* = 16 (9 males and 7 females) SCA1 patients. Apart from two sisters belonging to the same family, the other participants were not consanguineous. We also recruited 16 HC, without any history of neurological or psychiatric disorders or concurrent treatment with drugs acting on the central nervous system, who were strictly matched to patients 1:1 for age (± 1 year) and sex. The institutional Ethics Committee approved the study protocol (CE 453/2021), and all subjects gave their informed written consent. The study was conducted according to the Declaration of Helsinki and followed international research ethical principles involving human subjects.

### Neuropsychological testing

At enrollment, SCA1 patients were examined through an extensive neuropsychological battery including the following tests: Frontal Assessment Battery (FAB) [17], FAS for verbal fluency [18], Trail Making Test (TMT) [19], Raven Colored Progressive Matrices (RCPM) [20], Stroop Color and Word Interference Test (SCWT) [21], Rey-Osterrieth Complex Figure (ROCF) [22], and Babcock’s short tale (BST) [23]. The Emotion Attribution Task (EAT) [24] and Visual Analogue Test for Anosognosia for motor impairment (VATA-m) [25] were also administered to explore affective and self-awareness functions. We used Italian validated versions, and raw scores were adjusted for gender, age, and educational level according to established correction grids. Individual test performance was considered abnormal according to available cutoff scores.

### Electrophysiological assessment

**Fig. 1:**
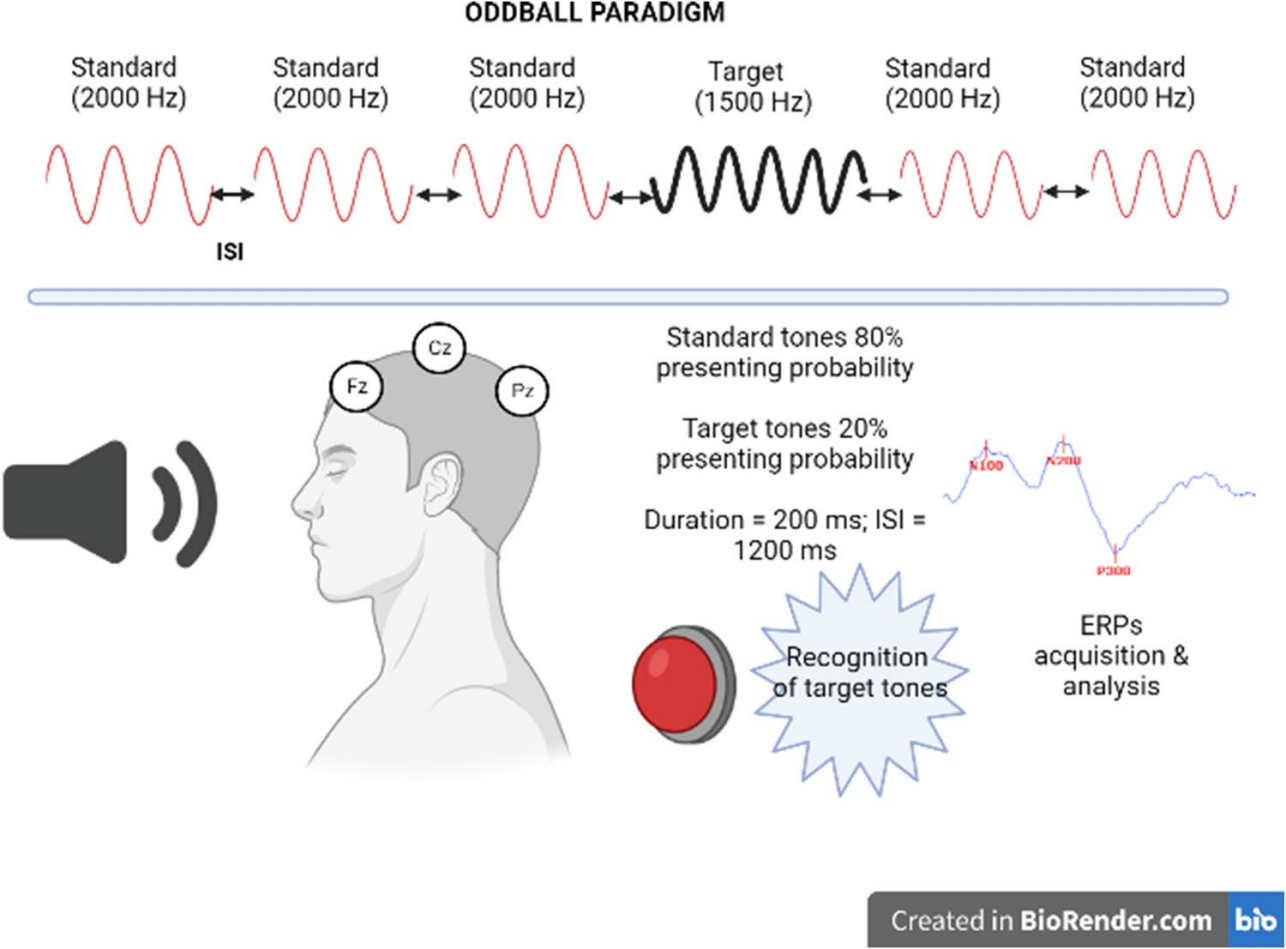
Schematic representation of the oddball paradigm employed for ERP acquisition

Within 1 month of clinical and neuropsychological evaluation, all patients underwent electrophysiological assessment for ERP analysis. Briefly, we used Keypoint^TM^ software (Natus Neurology Incorporated, Middleton, WI, USA) and administered auditory stimuli (average 74.97 ± 3.15 dB sound level to avoid uncomfortableness) using an oddball paradigm. The oddball paradigm consisted of at least 100 “standard” and “target” stimuli presented binaurally via earphones. Standard stimuli had 80% presenting probability and 2000-Hz frequency, whereas target stimuli had 20% presenting probability and 1500-Hz frequency. All tones were presented in a pseudo-randomized manner, had a 200-ms duration and an interstimulus interval of 1200 ms. In our experiment, subjects were instructed to concentrate on target stimuli: a brief practice trial was carried out to allow the correct discrimination between standard and target stimuli, and then subjects were asked to perform a simple motor task (e.g., clicking the pen tip) upon the onset of target tones. In the meantime, EEG signals were recorded from the scalp using Ag/AgCl electrodes at Fz, Cz, and Pz sites of the international 10/20 system, referred to linked earlobe electrodes. The ground electrode was located on the right hand, and impedance was kept below 5 kOhm. EEGs were filtered with a bandpass of 0.5–50 Hz and epochs were segmented at intervals of 100 ms pre-stimulus and 900 ms after-stimulus. Electrooculographic (EOG) recording was obtained, and artifact rejection was applied to remove trials with blinks (EOG voltage exceeding 75 μV) and other movements [26]. The responses to target and standard stimuli were averaged separately and at least two trials for each subject were conducted to prove the consistency of the waveforms. Peak latency and amplitude of components N100, N200, and P300 were measured in the averaged responses to target tones. We analyzed data recorded from the Cz electrode, where ERP components showed their maximum amplitude. N100, N200, and P300 components were identified. In the case of multi-peak morphology, the highest peak was chosen. When P300 subcomponents were recognizable, we considered for analysis the P3b, which better reflects the allocation of attentional resources for stimulus evaluation and memory updating [27]. Amplitude was determined using the baseline-to-peak method. The main steps of the employed paradigm are summarized in Fig. 1.

### Statistical analysis

Variables were expressed as counts and percentages when categorical and as mean ± standard deviation (SD) when continuous. The normality of data was assessed using the Shapiro-Wilk test. Differences between groups were analyzed through parametric Student’s *t*-test for independent samples or non-parametric Mann-Whitney *U* test as appropriate. Comparisons between categorical variables were assessed using Fisher’s exact test. A receiver operating characteristic (ROC) curve analysis was carried out to establish ERP components’ discriminatory power. The area under the curve (AUC) and significance values were obtained. AUC values interpretation was determined according to Mandrekar et al. [28]. The relationship between neuropsychological, clinical, and electrophysiological measures in the SCA1 group was assessed through Spearman correlation analysis. Partial correlation analysis was also employed to control for the potential confounding effect of cerebellar motor symptoms in the assessment of neuropsychological tests. The significance level was set to *p* < 0.05. Since this research was a pilot study, we did not apply any correction for multiple comparisons. All analyses were performed using SPSS Version 25 (IBM Corporation, Armonk, USA) and GraphPad Prism version 8 (GraphPad Software Inc., San Diego, USA).

## Results

**Fig. 2:**
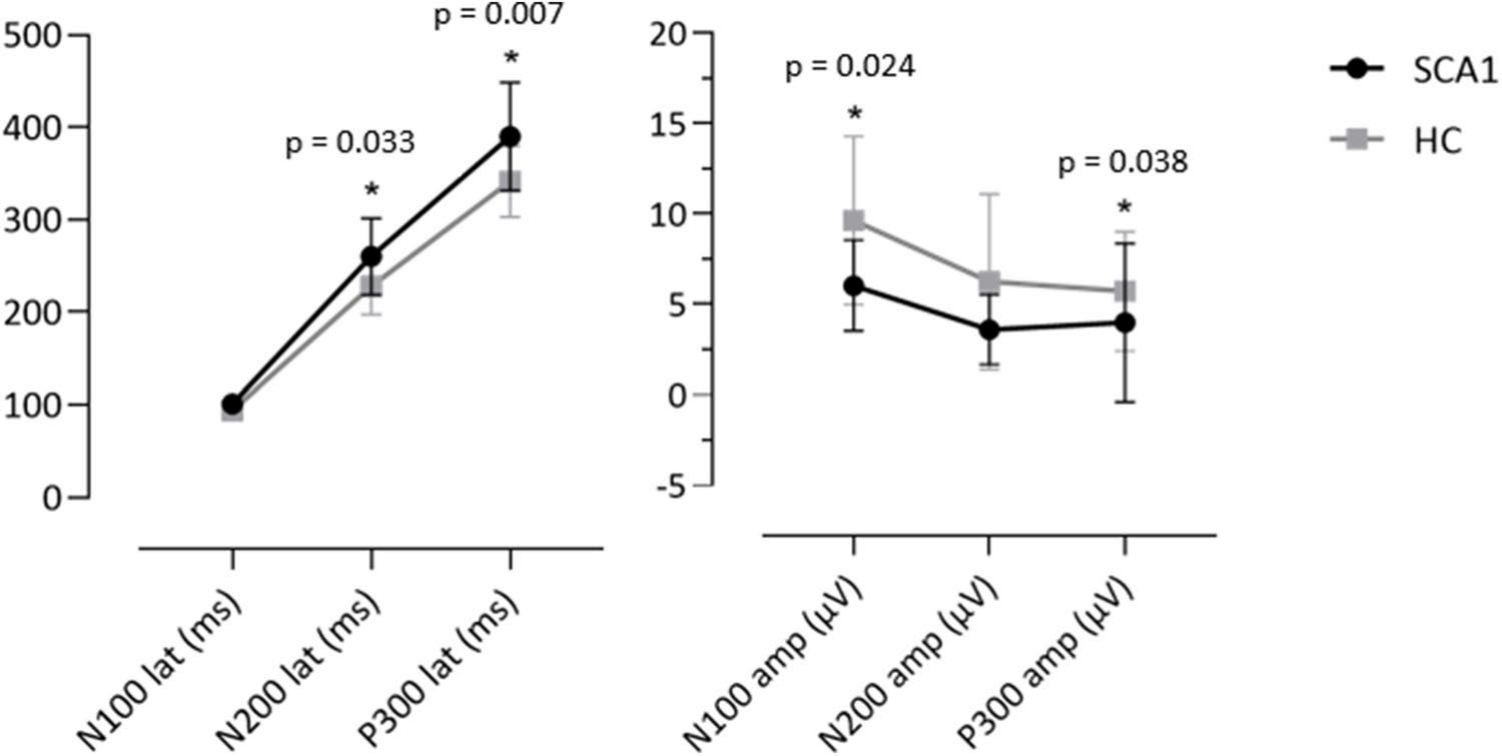
Means of latency and amplitude values of ERP components are plotted. Error bars represent standard deviations. Significant between-groups differences (*p*-value < 0.05) are highlighted with the asterisk

Complete clinical, demographic, and electrophysiological characteristics of SCA1 patients and matched HC are shown in Table 1. SCA1 patients were 47.69 ± 8.16 years old, with an average age at onset of 41.27 ± 8.50 years, average disease duration of 6.47 ± 3.62 years, and mean score of the SARA scale = 11.53 ± 5.14. There were no statistically significant differences regarding age, sex, and educational level between groups. Analysis of ERPs revealed in SCA1 patients significantly increased latencies of N200 and P300 (*p* = 0.033, *p* = 0.007) and decreased amplitudes of N100 and P300 (*p* = 0.024, *p* = 0.038) than HC; see Table 1 and Fig. 2. We then used ROC curve analysis to establish which ERP component could represent the most reliable electrophysiological signature of SCA1 patients and found that P300 latency showed the highest AUC (0.777, *p* = 0.007); see Table 2. Furthermore, we found moderate inverse correlations between the severity of SARA score and the amplitudes of P300 (*r* = −0.692, *p* = 0.003) and N100 (*r* = −0.621, *p* = 0.010), and between P300 latency and the number of CAG repeats in the expanded allele (*r* = −0.607, *p* = 0.048); see Supplementary Table S1.

**Table 1:** Demographic, clinical, and electrophysiological characteristics of SCA1 patients and healthy controls (HC)

	SCA1	HC	*p*-value
Number of subjects	16	16	
Sex (males/females)	9/7	9/7	1.0
Age (years), mean (SD)	47.69 (8.16)	47.12 (8.16)	0.848
Educational level (years), mean (SD)	12.50 (3.03)	13.31 (4.27)	0.540
Disease duration (years), mean (SD)	6.47 (3.62)	-	-
Age at onset (years), mean (SD)	41.27 (8.50)	-	-
SARA score, mean (SD)	11.53 (5.14)	-	-
Number of repeats expanded allele, mean (SD)	47.73 (6.44)	-	-
N100 latency (ms), mean (SD)	101.32 (6.32)	94.17 (10.09)	0.065
N100 amplitude (μV), mean (SD)	6.04 (2.51)	9.64 (4.65)	**0.024**
N200 latency (ms), mean (SD)	260.25 (41.32)	228 (30.68)	**0.033**
N200 amplitude (μV), mean (SD)	3.61 (1.93)	6.25 (4.84)	0.105
P300 latency (ms), mean (SD)	390.18 (58.33)	341.50 (38.43)	**0.007**
P300 amplitude (μV), mean (SD)	3.99 (4.37)	5.72 (3.29)	**0.038**

*SARA* Scale for the Assessment and Rating of Ataxia. Statistically significant *p*-values are highlighted in bold

**Table 2:** ROC curve analysis of ERP components for the discrimination between SCA1 patients and HC

ERPs	AUC (95% CI)	*p*-value
N100 latency (ms)	0.691 (0.502–0.881)	0.065
N100 amplitude (μV)	0.734 (0.554–0.915)	**0.024**
N200 latency (ms)	0.721 (0.534–0.907)	**0.033**
N200 amplitude (μV)	0.668 (0.478–0.857)	0.105
P300 latency (ms)	0.777 (0.612–0.942)	**0.007**
P300 amplitude (μV)	0.715 (0.531–0.898)	**0.038**

*ERPs* event-related potentials; *AUC* area under the curve. Statistically significant *p*-values are highlighted in bold

Regarding neuropsychological assessment, the average test scores of the whole cohort were within normal limits. Nonetheless, out of 16 subjects, some showed impaired performances in the following: FAB (12.5%), FAS (25%), TMT-A and TMT-B (18.7%), SCWT (6.2%), ROCF (12.5%), BST (6.2%), VATA-m (37.5%), and EAT (25%). Concerning the latter, we found that the recognition of fear and anger was mostly affected with error rates respectively of 62.5% and 75%. Significant correlations between neuropsychological test scores and the severity of cerebellar motor involvement were also observed (see Table 3). The relationship between neuropsychological and electrophysiological measures was then investigated using Spearman correlation analysis (see Supplementary Table S1). A moderate inverse correlation between P300 latency and EAT score (*r* = −0.633, *p* = 0.027) was found. Since we could not exclude the interference of dysarthria, dysmetria, and tremor in the assessment of some neuropsychological tests (namely FAB, FAS, SCWT, TMT-A, and TMT-B), SARA scores were used as a control variable in partial correlation analysis. Moderate correlations were confirmed between N200 latency and both FAB (*r* = −0.520, *p* = 0.047) and SCWT (*r* = 0.538, *p* = 0.039).

**Table 3:** Neuropsychological assessment in SCA1 patients and Spearman correlations with severity of motor scores

Test (cutoff score)	Score, mean (SD)	Number of patients (%) with pathological score	Correlation with SARA score, *r* (*p*)
MMSE (< 24)	29.33 (0.82)	0	0.180 (0.522)
FAB (< 13.48)	15.79 (1.37)	2 (12.5%)	−0.449 (0.081)
FAS fluency test (< 17.35)	22.90 (8.46)	4 (25%)	−**0.647 (0.009)**
TMT-A (> 94) TMT-B (> 283)	68.93 (28.04) 121.08 (36.99)	3 (18.7%) 3 (18.7%)	**0.664 (0.010)** **0.705 (0.007)**
RCPM (< 18.96)	30.75 (3.29)	0	−**0.549 (0.034)**
SCWT time (> 36.92)	21.57 (8.52)	1 (6.2%)	0.234 (0.384)
SCWT errors (> 4.24)	0.39 (0.85)	0	0.039 (0.896)
ROCF copy (< 28.87) ROCF recall (< 9.46)	33.79 (2.43) 13.16 (5.47)	0 2 (12.5%)	−0.347 (0.246) −0.248 (0.415)
BST (< 4.75)	8.87 (3.37)	1 (6.2%)	0.090 (0.751)
VATA-m (> 6.26)	6.00 (5.16)	6 (37.5%)	−0.174 (0.569)
EAT (< 44.19)	48.83 (8.49)	4 (25%)	−0.314 (0.321)

*SARA* Scale for the Assessment and Rating of Ataxia; *MMSE* Mini-Mental Status Examination; *FAB* Frontal Assessment Battery; *TMT* Trail Making Test; *RCPM* Raven Colored Progressive Matrices; *SCWT* Stroop Color and Word Test; *ROCF* Rey-Osterrieth complex figure test; *BST* Babcock’s short tale; *VATA-m* Visual Analogue Test assessing anosognosia for motor impairment; *EAT* Emotion Attribution Task. Higher scores indicate better performance on MMSE, FAB, FAS, RCPM, ROCF, BST, and EAT; higher scores indicate worse performances on TMT-A, TMT-B, SCWT, and VATA-m. *R*, correlation coefficient. Statistically significant *p*-values are highlighted in bold

## Discussion

This study provided an extensive neuropsychological and electrophysiological evaluation of a homogeneous cohort of SCA1 patients. Even though the overall cognitive performances were within normal limits, early differences in auditory ERPs could be detected in SCA1. Indeed, compared with HC, average latency values of N200 and P300 were increased and average amplitudes of N100 and P300 were decreased. We also found that, among ERP measures, P300 latency was the most useful electrophysiological marker in the discrimination of SCA1 from HC, whereas the severity of cerebellar motor symptoms significantly correlated with both P300 and N100 amplitudes. Lastly, P300 and N200 latency showed significant correlations with the scores obtained on EAT, FAB, and SCWT tests. Taken together, these findings suggest the early involvement in SCA1 patients of specific networks engaged in the generation of ERPs and their relationship with both motor and non-motor features.

The role of the cerebellum as a key component interacting with cortical areas in the control of attentional processes has been highlighted in previous literature: Mannarelli et al. studied the effects of transcranial direct current stimulation (tDCS) delivered over the left cerebellar hemisphere in 15 healthy participants and found that cathodal cerebellar tDCS significantly decreased neural excitability through hyperpolarization and reduced the amplitude of N100, N200, and P300 [29]. Hyperpolarization may suppress cerebellar-brain inhibition (CBI), a functional pathway promoting the coordination and synchronization of both motor and non-motor circuits through the interaction at different levels of the cerebellothalamocortical tract. Since the cerebellum is involved in the temporal and spatial tuning of cortical activity, inhibitory cathodal tDCS could promote the desynchronization of cortical neurons and the altered allocation of selective attentional resources from the early phase of stimulus perception to its discrimination [29]. Our findings are in line with previous research reporting significantly increased latency and decreased amplitude of auditory and visual P300 in chronic neurodegenerative disorders involving the cerebellum [14, 30]. On the other hand, in contrast with previous research [30], we found that N200 latency may also provide an acceptable AUC in detecting SCA1 and shows moderate correlations with frontal function scores. Even though N200 could be partly influenced by exogenous factors and is mainly involved in stimulus categorization, the role of this component in cognitive processing is being increasingly recognized. Bennys and colleagues reported that N200 is a feasible biomarker of cognitive deterioration in MCI and preclinical AD patients [31]. N200 latency prolongation has been also described in subcortical patterns of cognitive deterioration, i.e., Parkinson’s disease (PD) [32]. Tachibana and colleagues described ERPs during semantic discrimination tasks in six patients with pure cerebellar atrophy and two patients with olivopontocerebellar atrophy, reporting longer N200 latencies [33]. Concerning the earliest stages of information processing, decreased N100 amplitude was reported in PD [34], whereas N100 latency was increased in both cerebellar [30] and non-cerebellar disorders [35] and showed significant correlations with visual working memory tasks [36].

Intriguingly, in our study, P300 latency was the only neurophysiological measure correlating with EAT score. It was previously reported that anodal high-definition tDCS over the temporoparietal junction (one of the P300 generators) determined improvement in facial emotion processing performance in healthy subjects [37]. Furthermore, both the cerebellum and basal ganglia are involved in the cortical-subcortical networks underlying the recognition and discrimination of emotions. Notably, negative emotions are thought to activate phylogenetically older circuits (including the cerebellum) than positive ones, probably reflecting a defense system against potential threats [38], which is in line with the results of the present study. Even though research on non-invasive stimulation to improve emotional impairment in SCA1 patients is lacking, Ruggiero and colleagues observed in PD that anodal cerebellar tDCS enhances emotional recognition in response to sad facial expressions by about 16%, whereas no improvement was observed in the recognition of happiness and neutral facial expressions [39].

The abnormalities of auditory ERP components may provide valuable insights into the brain mechanisms underlying the neuropsychological dysfunction characteristic of SCAs. More in detail, an early decline of attention, fluency, executive, visuospatial, and emotional functions in some patients of our cohort was observed, in line with previous literature [40]. Prominent executive and attentional dysfunction were reported in several studies evaluating cognitive profile in SCA1 [41–45, 4, 5, 46], worse than other SCAs [42, 43], whereas deficits in visuospatial perception and memory have yielded conflicting results [5, 41, 46]. Different involvement of phonemic and semantic fluency has been observed as well [47, 44–46]. These discrepancies may arise from the analysis of heterogenous testing of cognitive functions and relatively small sample sizes, as summarized in Table 4. In the context of cerebellar involvement in cognitive control, a meta-analysis [38] reported that language and executive tasks activate regions of the Crus I and lobule VII (involved in prefrontal-cerebellar loops), whereas emotional processing involved the vermal lobule VII, which is implicated in cerebellar-limbic circuitry. Additionally, our results highlight the role of anosognosia, as this domain was impaired in 37.5% of SCA1 patients. Even though cognitive mechanisms of anosognosia remain uncertain, previous evidence suggested in AD a significant correlation between anosognosia scores and the atrophy of the cerebellar vermis [48] and an interplay between the cerebellum and the anterior default mode network in non-memory anosognosia [49], thus supporting the need for a thorough assessment of self-awareness in cerebellar patients. Nonetheless, no significant correlations were found between VATA-m scores and ERPs, thus suggesting the limited feasibility of these electrophysiological measures as biomarkers of anosognosia.

**Table 4:** Studies investigating the cognitive profile in SCA1 patients

Study	Patients	Age (years)	Disease duration (years)	Neuropsychological tests	Outcome
Bürk et al., 2001 [41]	14 SCA1	48.2 ± 9.3	9.6 ± 5.1	IQ, Digit Span, ROCF, WMS, Word lists, Verbal fluency, WCST	Preserved visuospatial memory and attention; verbal memory/executive dysfunction
Bürk et al., 2003 [42]	11 SCA1	48.6 ± 9.4	8.9 ± 5.4	IQ, MMSE, Digit Span, ROCF, WMS, Word lists, Verbal fluency, WCST	Executive dysfunction prominent in SCA1 compared with SCA2/3
Klinke et al., 2010 [43]	6 SCA1	45.7 ± 9.9	9.3 ± 6.4	SCT, SCR, ToH, Semantic and phonemic fluency, Response Inhibition, ICR, ICE, Verbal memory, Figural learning, MWT-B-IQ, BNT	Attentional and executive dysfunctions worse in SCA1 compared with SCA2/3/6
Sokolovsky et al, 2010 [47]	2 SCA1	36 ± 0	5.5 ± 0.7	VOSP, GDAT, GNT, RMW, RMF, WMS, TEA, WAIS-R, NART, Verbal fluency, Hayling test, SCWT, MCST	Relatively intact profiles in SCA1 compared with SCA7/2/3; 1 SCA1 patient with Theory of Mind deficit
Orsi et al., 2011 [44]	6 SCA1	45 ± 8	8 ± 5	MMSE, Digit and Corsi span, SCWT, WAIS Arithmetic, BSRT, PWRT, SSRT, RWLR, UWLR, BVR, JLO, ROCF, WAIS-BD, TOL, WCST, SCWT, Phonemic fluency, Semantic fluency	Homogeneous profile among SCA subgroups (SCA 1/2/6/8) with a prominent role of attention, memory, and executive functions
Fancellu et al., 2013 [45]	20 SCA1	47.4 ± 11.4	9.3 ± 5.2	MMSE, Digit span, FCS, Attentional matrices, mWCST, RCPM, Phonemic fluency, Semantic fluency, Benton test	Deficits in executive functions (phonemic and semantic fluency, attentional matrices)
Ma et al., 2014 [4]	8 SCA1	47.0 ± 9.6	10.0 ± 4.9	Digit span, RVR, immediate and delayed recall (memory), SCWT, CDT, LTT, Orientation	Impairment of executive function, visuospatial perception, and attention
Moriarty et al., 2016 [5]	2 SCA1	36 ± 0	6.5 ± 0	Verbal fluency, Hayling test, SCWT, Symbol digit, Elevator counting, RMW, RMF	Deficits in executive functions, speed, attention, visual memory, and Theory of Mind; decline over time more rapid in SCA1 than SCA2/3/6/7
Nigri et al., 2022 [46]	11 SCA1, 14 *Pre*SCA1	44.3 (33.8–51.1) SCA1; 29.7 (18.7–50.3) *Pre*SCA1	6.0 (2.1–16.3)	MMSE, Digit span, SDMT, Phonemic fluency, Semantic fluency, ROCF, Calculation test	Compared with healthy controls, SCA1 had lower scores on MMSE, SDMT, phonemic fluency, and ROCF copy test; *Pre*SCA1 subjects did not differ except for SDMT

*IQ* intelligence quotient; *ROCF* Rey-Osterrieth Complex Figure; *WMS* Wechsler Memory Scale; *WCST* Wisconsin Card-Sorting; *MMSE* Mini-Mental State Examination; *SCT* Symbol counting test; *SCR* Symbol+choice reaction; *ToH* Tower of Hanoi; *ICR* Inverse choice reaction; *ICE* Inverse choice errors; *MWT-B-IQ* Mehrfachwahl Wortschatz Interferenztest; *BNT* Boston naming test; *VOSP* Visual Object and Space Perception battery; *GDAT* Graded Difficulty Arithmetic Test; *GNT* Graded Naming Test; *RMW* Recognition Memory test for Words; *RMF* Recognition Memory test for Faces; *TEA* Test of Everyday Attention; *WAIS-R* Wechsler Adult Intelligence Scale-Revised; *NART* National Adult Reading Test; *SCWT* Stroop Color and Word Test; *MCST* Modified Card Sorting Test; *BSRT* Buschke Selective Reminding Test; *PWRT* Paired Word Recall test; *SSRT* Short Stories Recall test; *RWLR* Related Word List Recognition; *UWLR* Unrelated Word List Recognition; *BVR* Benton Visual Retention; *JLO* Judgment of Line Orientation; *WAIS-BD* WAIS Block Design; *TOL* Tower of London; *FCS* Forward Corsi span; *mWCST* modified Wisconsin Card-Sorting; *RCPM* Raven Colored Progressive Matrices; *RVR* Rapid Verbal Retrieve; *CDT* Clock Drawing Test; *LTT* logical thinking test; *SDMT* Symbol Digit Modalities Test. Variables are expressed as mean ± standard deviation or median (min–max range)

To the best of our knowledge, this is the first research providing an extensive characterization of ERPs as early neurophysiological markers of cognitive dysfunction in a homogenous cohort of SCA1 patients. Our results also support the multiple system involvement in SCA1: on one side, we can hypothesize that cerebellar degeneration can likely alter P300 as a result of the dysfunction of cortico-cerebellar circuits; on the other, the key role of early components (N100, N200) suggests the participation of sensory pathways involved in the discrimination and categorization of auditory stimuli. Some limitations of the present study should be mentioned as well, such as (i) the unavailability of neuroimaging features that could have clarified any anatomical correlates of cognitive function; (ii) the lack of assessment of cognitive-affective functions in HC; (iii) the use of a neuropsychological battery with several ataxia-dependent tests; (iv) the cross-sectional design and small sample size, which require a cautious interpretation of these results; (v) the use of a simple auditory oddball paradigm, which may not be sufficient to explain complex cognitive processes. Nonetheless, several issues have been addressed: firstly, we carefully assessed the relationship between ataxia severity and neuropsychological test scores in partial correlation analysis, thus limiting the influence of motor impairment on cognitive outcomes. Secondly, even though the small number of patients and controls limited the power of statistical tests, the electrophysiological assessment was conducted through a strict matching procedure, thus controlling for the effect of age and gender in ERP components. Lastly, even though the cross-sectional design has several weaknesses, SCA1 patients involved in the present study are currently enrolled in a longitudinal protocol to further elucidate the usefulness of ERPs as biomarkers of cognitive dysfunction progression.

## Conclusions

This pilot study highlights the feasible role of ERPs as neurophysiological markers of cognitive and emotional processing in SCA1 patients. Future studies, with prospective designs and involving larger cohorts, are therefore warranted to confirm these preliminary findings and further elucidate the underlying mechanisms of CCAS.

## Supplementary Information

ESM 1 (DOCX 18 kb)

## Data Availability

Anonymized data can be obtained upon reasonable request from qualified researchers.
