# Supplementary material for: Electrophysiological and neuropsychological assessment of cognition in spinocerebellar ataxia type 1 patients: a pilot study
Source: Neurol Sci. 2023 Jan 14;44(5):1597–606. doi: 10.1007/s10072-022-06597-5 (PMC10102071; doi:10.1007/s10072-022-06597-5)
Supplement: Supplementary file 1 — (DOCX 18 kb) [file 10072_2022_6597_MOESM1_ESM.docx]

**Supplementary material**

**Supplementary Table S1:** Spearman and partial correlation analysis between clinical characteristics, motor and cognitive-affective test scores, and ERP components.

| Variables | N100 lat (r,p) | N100 amp (r,p) | N200 lat (r,p) | N200 amp (r,p) | P300 lat (r,p) | P300 amp (r,p) |
| --- | --- | --- | --- | --- | --- | --- |
| Age, years | - 0.027, 0.884 | 0.188, 0.302 | 0.219, 0.228 | 0.087, 0.637 | 0.271, 0.133 | 0.109, 0.554 |
| Sex | 0.191, 0.295 | - 0.287, 0.112 | - 0.239, 0.188 | - 0.027, 0.882 | - 0.341, 0.056 | - 0.130, 0.479 |
| Number of repeats expanded allele | 0.239, 0.479 | - 0.389, 0.236 | - 0.124, 0.716 | - 0.207, 0.542 | **- 0.607, 0.048** | - 0.097, 0.777 |
| Age at onset, years | 0.079, 0.780 | 0.295, 0.287 | 0.044, 0.877 | 0.349, 0.203 | 0.055, 0.844 | 0.150, 0.593 |
| Disease duration, years | - 0.284, 0.306 | - 0.325, 0.238 | 0.269, 0.333 | 0.182, 0.515 | - 0.190, 0.498 | - 0.132, 0.639 |
| SARA | 0.221, 0.410 | **- 0.621, 0.010** | 0.405, 0.119 | 0.353, 0.179 | 0.067, 0.807 | **- 0.692, 0.003** |
| MMSE | - 0.409, 0.147 | - 0.419, 0.135 | 0.010, 0.973 | 0.048, 0.872 | 0.319, 0.267 | 0.085, 0.773 |
| FAB* | 0.042, 0.886 | 0.124, 0.672 | **- 0.520, 0.047** | 0.221, 0.448 | - 0.154, 0.599 | 0.188, 0.520 |
| FAS* | 0.216, 0.458 | 0.213, 0.464 | 0.038, 0.898 | 0.427, 0.052 | 0.241, 0.406 | - 0.299, 0.300 |
| TMT-A* | 0.099, 0.749 | 0.066, 0.831 | - 0.090, 0.770 | 0.082, 0.789 | 0.252, 0.406 | - 0.155, 0.614 |
| TMT-B* | - 0.164, 0.611 | - 0.224, 0.485 | - 0.067, 0.835 | - 0.197, 0.539 | - 0.064, 0.805 | - 0.266, 0.403 |
| RCPM | 0.117, 0.677 | 0.394, 0.146 | - 0.306, 0.267 | - 0.158, 0.575 | - 0.367, 0.178 | 0.223, 0.424 |
| SCWT time* | 0.125, 0.684 | - 0.053, 0.865 | **0.538, 0.039** | - 0.003, 0.991 | 0.370, 0.214 | - 0.131, 0.669 |
| SCWT errors* | 0.293, 0.331 | 0.024, 0.939 | - 0.339, 0.258 | 0.080, 0.796 | - 0.365, 0.220 | - 0.095, 0.757 |
| ROCF copy | 0.227, 0.456 | 0.269, 0.374 | - 0.495, 0.086 | - 0.402 0.173 | - 0.057, 0.854 | - 0.091, 0.769 |
| ROCF recall | 0.058, 0.851 | 0.029, 0.925 | - 0.342, 0.253 | 0.129, 0.673 | 0.361, 0.226 | 0.041, 0.893 |
| BST | 0.134, 0.634 | 0.120, 0.671 | 0.327, 0.234 | - 0.114, 0.634 | - 0.104, 0.713 | - 0.306, 0.268 |
| VATA-m | 0.231, 0.447 | 0.022, 0.942 | 0.350, 0.242 | 0,186, 0.542 | 0.236, 0.437 | 0.031, 0.921 |
| EAT | - 0.353, 0.260 | 0.324, 0.304 | - 0.400, 0.198 | - 0.424, 0.169 | **- 0.633, 0.027** | 0.269, 0.398 |

*SARA:* Scale for the Assessment and Rating of Ataxia; *MMSE*: Mini-Mental Status Examination; *FAB*: Frontal Assessment Battery; *TMT*: Trail Making Test; *RCPM*: Raven Colored Progressive Matrices; *SCWT*: Stroop Color and Word Test; *ROCF*: Rey-Osterrieth complex figure test; *BST*: Babcock’s short tale; *VATA-m*: Visual Analogue Test assessing anosognosia for motor impairment; *EAT*: Emotion Attribution Task. Higher scores indicate better performance on MMSE, FAB, FAS, RCPM, ROCF, BST, and EAT; higher scores indicate worse performances on TMT-A, TMT-B, SCWT, and VATA-m. Partial correlation analyses controlling for the effect of SARA score are indicated with the asterisk. R=correlation coefficient. Statistically significant *p*-values are highlighted in bold.
